# Effect of *Lactobacillus acidophilus* D2/CSL (CECT 4529) supplementation in drinking water on chicken crop and caeca microbiome

**DOI:** 10.1371/journal.pone.0228338

**Published:** 2020-01-24

**Authors:** Alessandra De Cesare, Claudia Sala, Gastone Castellani, Annalisa Astolfi, Valentina Indio, Alberto Giardini, Gerardo Manfreda

**Affiliations:** 1 Department of Agricultural and Food Sciences, Alma Mater Studiorum - University of Bologna, Ozzano dell’Emilia, Bologna, Italy; 2 Department of Physics and Astronomy, University of Bologna, Bologna, Italy; 3 Centro Interdipartimentale di Ricerche sul Cancro "Giorgio Prodi" (CIRC), Bologna, Italy; 4 Centro Sperimentale del Latte, Zelo Buon Persico, Lodi, Italy; Tokat Gaziosmanpasa University, TURKEY

## Abstract

In this study we gained insights into the effects of the supplementation with *Lactobacillus acidophilus* D2/CSL (CECT 4529) in the chicken drinking water on crop and caeca microbiomes. The probiotic was supplemented at the concentrations of 0.2 g *Lactobacillus acidophilus*/day/bird and 0.02 g *Lactobacillus acidophilus*/day/bird and its effect on the crop and caeca microbiomes was assessed at 14 and 35 days of rearing. The results showed that mean relative abundance of *Lactobacillus acidophilus* in the caeca did not show significative differences in the treated and control birds, although *Lactobacillus acidophilus* as well as *Faecalibacterium prausnitzii*, *Lactobacillus crispatus* and *Lactobacillus reuteri* significantly increased over time. Moreover, the treatment with the high dose of probiotic significantly increased the abundance of *Clostridium asparagiforme*, *Clostridium hathewayi* and *Clostridium saccharolyticum* producing butyrate and other organic acids supporting the chicken health. Finally, at 35 days, the Cell division protein FtsH (EC 3.4.24.-) and the Site-specific recombinase genes were significantly increased in the caeca of birds treated with the high dose of probiotic in comparison to the control group. The results of this study showed that *Lactobacillus acidophilus* D2/CSL (CECT 4529) supplementation in the drinking water at the concentrations of 0.2 and 0.02 g *Lactobacillus acidophilus*/day/bird improved beneficial microbes and functional genes in broiler crops and caeca. Nevertheless, the main site of action of the probiotic is the crop, at least in the early stage of the chicken life. Indeed, at 14 days *Lactobacillus acidophilus* was significantly higher in the crops of chickens treated with the high dose of LA in comparison to the control (14.094 vs 1.741%, p = 0.036).

## Introduction

Probiotics are classified as live non-pathogenic microorganisms that are capable of maintaining a normal gastrointestinal microbiota [1, 2]. They can contain one or many strains of microbial species, with the more common ones belonging to the genera *Lactobacillus*, *Bifidobacterium*, *Enterococcus*, *Bacillus* and *Pediococcus* [3]. The primary function of the gastrointestinal tract is to digest and absorb nutrients and a well-balanced microbiota is crucial for optimal animal health and performance [4].

Presently there is a great deal of interest in the possibility of altering the intestinal microbiota in a beneficial and “natural” way to improve animal health thus preventing the need to use antibiotics. Indeed, the increasing incidence of antibiotic resistance is considered to be one of the greatest threats to public health globally. Feeding broilers with probiotic *Lactobacilli* is potentially a useful approach to address this concern. *Lactobacilli* become established in the gastro-intestinal (GI) tract of chicks soon after hatching and their metabolic activity lowers the pH of the digesta, which in turn inhibits the proliferation of enterobacteria and other unwanted bacteria [5, 6].

Ideally, researchers select the promising probiotic strains from the indigenous intestinal microbiota by supposing that these microorganisms have a symbiotic relationship with the host, so they could colonize the GI tract. Modes of action of probiotic *Lactobacilli* include competitive exclusion toward harmful bacteria, alteration of microbial and host metabolism, and immunity modulation [1, 7–11]. The bacterial strain, the dosage (i.e., colony forming unit (cfu)/bird/day), the duration of the treatment and the delivery strategy are among the critical factors influencing the probiotics efficacy. There are many different methods for administering probiotic preparations to broiler chickens. They are mainly represented by supplementation to the feed or water, through gavage (including droplet or inoculations), spraying or via application to the litter. However, adding the probiotic to the feed is the most commonly used method in poultry production [12]. In contrast, it is known that introducing probiotics through drinking water, into the crop by tube and syringe, with crumbles, or by spraying on the bird environment and litter had no effect on their survival rate [13–15].

In a previous study [16] we evaluated the effects of supplementing *Lactobacillus acidophilus* D2/CSL (CECT 4529) (LA) in broiler chicken feed on production performance, foot pad dermatitis and caecum microbiome. The results showed that supplementation with *Lactobacillus acidophilus* D2/CSL (CECT 4529) at the recommended dietary dosage of 1x10^9^ cfu/kg of feed significantly improved body weight at 28 days (commercial weight of 1.5 kg) and feed conversion rate from 0 to 41 days, while reducing the incidence of pasty vent. *Faecalibacterium prausnitzii* and *Subdonigranulum variabile*, which are *Clostridium* cluster IV microorganisms producing primarily butyrate, were not significantly different between treated and control groups and the relative abundance of *Lactobacillus acidophilus* in the caeca of treated chickens was comparable with that of the control group, although a positive effect of supplementing *Lactobacillus acidophilus* was observed in regards to functional genes as β-glucosidase and improving animal production performance and health.

In the present study we explored the effect of supplementing two concentrations of probiotic LA in the drinking water on the chicken crop and caeca microbiome sampled at day 1, 14 and 35.

## Materials and methods

### Animals and treatments

The experiment was approved by the Ethical Committee of the University of Bologna on 17/3/2014 (ID: 10/79/2014). A total of 120 day-old male Ross 308 chicks, obtained from the same breeder flock and hatching session, were used. Birds were vaccinated against infectious bronchitis virus, Marek’s disease virus, Newcastle and Gumboro diseases and coccidiosis at the hatchery. At the housing time, chicks were divided into three separate rooms, labelled as A, B and C, containing two pens each (20 birds/pen). The pens were equipped with pan feeders to assure at least 2 cm/bird of front space and an independent drinking system with 1 nipple/5 birds. Feed and not chlorinated water were provided *ad libitum*. The basal diet composition is given in Table 1. The experiment lasted 35 days. Photoperiod and temperature programs were set up according to the European welfare regulation 43/2007 [17]. The chicks housed in room A drank approximately 0.2 g of probiotic each once a day. This concentration of LA was defined as high (HD) and corresponded to 10^10^ colony forming unit (CFU) of LA/bird/day. The chicks housed in room B drank approximately 0.02 g of probiotic once a day. This concentration of LA was defined as low (LD) and corresponded to 10^9^ CFU of LA/bird/day. Finally, the chicks housed in room C drank water without supplementation of probiotic. To be able to provide LA to chickens in the drinking troughs, the latter were taken out for 1.5 hours every morning and supplemented again with or without the LA in drinking water. When the water with or without the probiotic was supplemented after the 1.5 hour suspension period all the birds drank from the drinking trough and we assumed that they ingested the targeted dose of treatment. The probiotic strain *L*. *acidophilus* D2/CSL was isolated from the GI tract of a healthy adult chicken [18] and supplied by Centro Sperimentale del Latte S.r.l. (Lodi, Italy).

**Table 1:** Basal diets composition.

	Starter (0–14 d)	Grower (15–28 d)	Finisher (29–41 d)
**Ingredients, g/100 g**			
Corn	42.17	34.96	12.73
White corn	0.00	0.00	15.00
Wheat	10.00	20.00	25.01
Sorghum	0.00	0.00	5.00
Soybean meal	23.11	20.63	17.60
Expanded soybean	10.00	10.00	13.00
Sunflower	3.00	3.00	3.00
Corn gluten meal	4.00	3.00	0.00
Soybean oil	3.08	4.43	5.48
Dicalcium phosphate	1.52	1.20	0.57
Calcium carbonate	0.91	0.65	0.52
Sodium bicarbonate	0.15	0.10	0.15
Salt	0.27	0.27	0.25
Choline chloride	0.10	0.10	0.10
Lysine sulphate	0.59	0.55	0.46
Dl-methionine	0.27	0.29	0.30
Threonine	0.15	0.14	0.14
Xylanase	0.08	0.08	0.08
Phytase	0.10	0.10	0.10
Vitamin-mineral premix^1^	0.50	0.50	0.50
**Proximate composition, g/100g**			
Dry matter	88.57	88.65	88.64
Protein	22.70	21.49	19.74
Lipid	7.06	8.24	9.74
Fiber	3.08	3.04	3.07
Ash	5.85	5.17	4.49
ME (kcal/kg)	3,076	3,168	3,264

^1^ Provided the following per kg of diet: vitamin A (retinyl acetate), 13,000 IU; vitamin D3 (cholecalciferol), 4,000 IU; vitamin E (DL-α_tocopheryl acetate), 80 IU; vitamin K (menadione sodium bisulfite), 3 mg; riboflavin, 6.0 mg; pantothenic acid, 6.0 mg; niacin, 20 mg; pyridoxine, 2 mg; folic acid, 0.5 mg; biotin, 0.10 mg; thiamine, 2.5 mg; vitamin B_12_ 20 μg; Mn, 100 mg; Zn, 85 mg; Fe, 30 mg; Cu, 10 mg; I, 1.5 mg; Se, 0.2 mg; ethoxyquin, 100 mg.

### Sample collection for metagenomics

To characterize the impact of the supplementation of LA at the difference concentrations in drinking water on the crop and caecum microbiome, representing both the microbial populations and the genes related to their metabolic functions, 5 animals were randomly selected and humanely euthanized at the arrival, before the LA application (day 1); 18 chickens (6/room and 3/pen) were randomly selected and humanely euthanized at day 14; 30 chickens (10/room and 5/pen) at the end of the rearing cycle at day 35. The entire gastrointestinal (GI) tract of each bird was dissected out and a small sample (i.e., 0.5 to 2 g) of crop as well as cecum content was collected into 2 ml sterile plastic tubes, flash freezed in liquid nitrogen and then stored at -80°C until further testing.

### DNA extraction and sequencing

The DNA was extracted from each sample of caecum and crop content as previously described [16]. Total DNA from each sample was fragmented and tagged with sequencing adapters using the Nextera XT DNA Library Preparation Kit (Illumina, San Diego, CA) and 82 extracted DNA samples resulted in libraries of the appropriate size and concentration to be sequenced. Shotgun metagenomic sequencing was performed using the HiScanSQ sequencer (Illumina) at 100 bp in paired-end mode.

### Bioinformatic and statistical analysis

Following sequencing, all reads were assessed for quality parameters and the paired end merged. The MG-RAST pipeline [19] (metagenomics.anl.gov) was used to identify the relative abundances of bacterial taxa performing a BLAST similarity search for the longest cluster representative against the M5rna database, integrating SILVA [20], Greengenes [21] and RDP [22]. Moreover, the sequenced reads were assigned to functional groups using the Kyoto Encyclopedia of Genes and Genome (KEGG) database (www.genome.jp/kegg/) [23] and their percentage of abundance was calculated.

Statistical analysis was performed separately for the crop and caeca samples on both taxonomic and functional genes.

The effect of treatment and time over the abundance of taxonomic or functional genes was evaluated using the package phyloseq 1.26.0 in R 3.5.1. In particular, DESeq was used to compute a Negative Binomial Generalized Linear Model and to test for significance of coefficients. The model was applied for the time interval between 14 and 35 days considering as covariates the treatment, the sampling time as well as their interaction meaning whether treatment effects were affected by time. The p-values, assessing the statistical significance of the variables, were adjusted for multiple testing using the Benjamini-Hochberg procedure. For each intestinal tract, DESeq was also used to perform a Likelihood Ratio Test at 14 days and 35 days to evaluate the effect of treatment on both the taxonomic and functional genes abundances. Moreover, pairwise comparisons between the three treatments (control, low dose and high dose of probiotic) were performed using the Wald test. Also in these cases, p-values were adjusted for multiple testing using the Benjamini-Hochberg procedure.

Alpha diversity was computed using an in-house pipeline that computes the indices from the normalized read counts. Alpha diversities of different groups were compared using the Student’s t-test. P-values were adjusted for multiple testing using the Benjamini–Hochberg procedure. In all statistical analysis, p values < 0.05 were considered statistically significant. Finally, Bray-Curtis beta diversity and Principal Coordinate analysis were computed using the scikit-bio 0.4.2 library in python 3.6.3.

## Results

### Metagenomic results

Metagenomic sequencing yielded an average of 5.706 million mapped reads/sample, with a Phread quality score always higher than 30. The 82 metagenomes sequenced are available from MG RAST (http://metagenomics.anl.gov/linkin.cgi?project=13081). The metagenome IDs are described in S1 Table.

### Taxonomic composition of caeca and crop microbiome

#### Phyla identified in the caeca and crop microbiome

At 14 and 35 days, the most abundant phyla did not show any significant differences in the caeca of birds belonging to the three test groups (i.e., high dose, low dose and control) (S2 Table). The only exception was the Actinobacteria at 35 days which showed a mean relative abundance (MRA) significantly lower in the low dose group compared to the control group (3.046 vs 4.189%, p = 0.017). The same result was observed in the caeca of birds belonging to the high dose group in comparison to the control (3.012 vs 4.189%, p = 0.005). In contrast, the MRA of Actinobacteria in the caeca of birds fed with the low and high dose of probiotic were not significantly different (S2 Table). The covariate results including treatment, sampling time and their interaction showed significant time (p = 0.003) and time by treatment (p = 0.021) interaction for the MRA of Actinobacteria found in the caeca of the tested groups, which significantly increased between 14 and 35 days. In comparison, the MRA of Bacteroidetes significantly decreased between 14 and 35 days due to both time (p = 0.008) and time and treatment interactions (p<0.000). In the crops of chickens fed the low dose probiotic, the MRA of Firmicutes at 14 days was significantly lower in comparison to the control group (77.768 vs 91.923%, p = 0.020) but this difference was not detected at 35 days (S2 Table). At this sampling time, Actinobacteria were significantly higher in the caeca of birds treated with the high dose of probiotic in comparison to the low dose group (7.156 vs 0.653%, p = 0.048), whereas Proteobacteria were significantly higher in the crops of birds receiving the low dose in comparison to the high dose treatment (13.945 vs 1.964%, p = 0.048). Between 14 and 35 days both Actinobacteria and the Firmicutes populations significantly increased in the crops of all tested treatments as influenced by time (p<0.000 and p = 0.044, respectively).

#### Classes identified in the caeca and crop microbiome

Clostridia, Bacilli, Bacteroidia, Erysipelotrichi, Actinobacteria, Gammaproteobacteria and Negativicutes were the most abundant bacterial classes identified in the caeca and crops (S3 Table). The class Actinobacteria at 35 days showed a MRA in the caeca significantly lower in the low dose group in comparison to the control group (3.059 vs 4.206%, p = 0.032). The same result was observed in the caeca of birds belonging to the high dose group in comparison to the control (3.025 vs 4.206%, p = 0.009). In the crops the same class at 35 days was significantly higher in the high dose group in comparison to the low dose group (7.154 vs 0.653%, p = 0.039). The covariate results showed that Actinobacteria and Bacilli significantly increased as effect of time in the tested groups, both in the caeca (p = 0.005 and p<0.000) and in the crops (p<0.000 and p = 0.050), whereas both Bacteroidia and Negativicutes significantly decreased in the caeca (p = 0.023 and 0.003) and only Negativicutes in the crops (p = 0.001).

#### Orders identified in the caeca and crop microbiome

Clostridiales, Bacteroidales, Lactobacillales, Bacillales, Erysipelotrichales, Selenomonadales, Enterobacteriales, Actinomycetales, Coriobacteriales and Xanthomonadales were the most abundant orders identified in the caeca and crops of the tested chickens (S4 Table). At 35 days Coriobacteriales had a MRA in the caeca significantly lower in both the low and high dose groups compared to the control group (1.449 vs 2.087%, p = 0.002) (1.450 vs 2.087%, p<0.000). Moreover, at 35 days Actinomycetales were significantly higher in the crops of the high dose group compared to the low dose group (6.980 vs 0.508%, p = 0.026). The covariate results showed that Lactobacillales significantly increased by time (p = 0.001 and 0.036) in both the caeca and crops of tested animals. Conversely, Selenomonadales significantly increased in the caeca (p = 0.001) whereas in the crops they significantly decreased (p<0.000). In the caeca Coriobacteriales significantly increased (p = 0.003) whereas Bacteroidales significantly decreased (p = 0.009). Finally, in the crops, Actinomycetales significantly increased (p<0.000), whereas Bacillales and Xanthomonadales significantly decreased (p = 0.003 and p<0.000).

#### Families identified in the caeca and crop microbiome

Lachnospiraceae, Eubacteriaceae, Bacteroidaceae, Erysipelotrichaceae, Clostridiaceae, Ruminococcaceae, Bacillaceae, Lactobacillaceae, Streptococcaceae, Enterobacteriaceae, Enterococcaceae, Veillonellaceae and Xanthomonadaceae were the 13 most abundant families identified in the caeca and crops of the tested birds. Moreover, Coriobacteriaceae, Peptococcaceae and Thermoanaerobacteraceae were detected in the caeca, whereas Staphylococcaceae and Corynebacteriaceae in the crops (S5 Table). At 35 days both Coriobacteriaceae and Lactobacillaceae showed a MRA in the caeca significantly lower in the low and high dose groups in comparison to the control groups (LD: 1.507 vs 2.180%, p = 0.001; 2.503 vs 6.138%, p = 0.048) (HD; 1.509 vs 2.180%, p = 0.001; 1.831 vs 6.138%, p = 0.002). In the crops, at 14 days Lactobacillaceae were significantly lower in the birds belonging to the low dose group in comparison to the control (56.940 vs 81.024%, p = 0.042). Conversely, Staphylococcaceae were significantly higher in the low dose group in comparison to the control (0.194 vs 0.182%, p = 0.045). At 14 days Enterococcaceae and Streptococcaceae were significantly lower in the birds belonging to the low dose group in comparison to both the control (0.663 vs 1.313, p = 0.003; 2.166 vs 4.707%, p = 0.037) and the high dose group (0.663 vs 1.173%, p = 0.041; 2.166 vs 8.097%, p = 0.029). Later on, at 35 days both Corynebacteriaceae and Staphylococcaceae were significantly higher in the crops of birds belonging to the high dose group (i.e., 3.653 and 3.118). The covariate analysis showed that Bacteroidaceae significantly decreased in the caeca of the tested groups between 14 and 35 days (p = 0.008), whereas Coriobacteriaceae, Lactobacillaceae, Peptococcaceae and Thermoanaerobacteraceae significantly increased over time (p = 0.005, <0.000, 0.035, 0.039). Beside time, the abundance of Lactobacillaceae was significantly affected by the interaction between time and treatment (p = 0.043). In the crops Lactobacillaceae and Staphylococcaceae significantly increased between 14 and 35 days (p = 0.029, p<0.000), whereas Veillonellaceae and Xanthomonadaceae significantly decreased (p<0.001 and p<0.001). Moreover, within the low dose group Enterococcaceae significantly decreased due to treatment (p = 0.045).

#### Genera identified in the caeca and crop microbiome

*Faecalibacterium*, *Ruminococcus*, *Eubacterium*, *Bacteroides*, *Clostridium*, *Subdoligranulum*, *Blautia*, *Lactobacillus*, *Bacillus*, *Streptococcus*, *Enterococcus*, *Escherichia* and *Shigella* were the 13 most abundant genera identified in both caeca and crops of the tested animals (Table 1). Moreover, the genera *Roseburia*, *Butyrivibrio*, *Ethanoligenens*, *Holdemania*, *Anaerotruncus*, *Desulfitobacterium*, *Dorea*, *Coprococcus* and *Eggerthella* were identified only in the caeca, while *Staphylococcus*, *Corynebacterium*, *Xanthomonas* and *Klebsiella* only in the crops (Table 2).

**Table 2:** Genera identified in the caeca and crops with a MRA (%) > 1 in at least one treatment (i.e., day 1, high dose (HD) 14 and 35 days, low dose (LD) 14 and 35 days, control (C) 14 and 35 days).

	Mean relative abundance (%) (standard error)						
	Day 1	HD 14d	HD 35d	LD 14d	LD 35d	C 14d	C35 d
Caeca							
*Faecalibacterium*	3.272 (0.917)	1.8 (0.088)	9.827 (1.251)	1.762 (0.066)	9.08 (1.756)	2.159 (0.136)	5.617 (0.714)
*Ruminococcus*	5.904 (0.86)	8.995 (0.487)	7.237 (0.2)	10.173 (0.991)	7.807 (0.336)	9.072 (0.999)	8.092 (0.537)
*Eubacterium*	4.541 (0.834)	7.153 (0.196)	6.629 (0.08)	7.309 (0.27)	6.67 (0.101)	7.071 (0.349)	6.489 (0.256)
*Bacteroides*	3.795 (0.528)	7.824 (0.792)	5.911 (0.211)	6.806 (0.877)	5.445 (0.303)	7.427 (0.865)	5.539 (0.419)
*Clostridium*	15.108 (2.482)	30.129 (1.687)	26.298 (0.711)	29.813 (0.829)	25.812 (0.845)	28.04 (1.594)	23.775 (0.676)
*Subdoligranulum*	2.461 (0.694)	1.723 (0.144)	2.963 (0.206)	1.513 (0.076)	3.947 (0.474)	5.176 (2.221)	4.588 (1.16)
*Blautia*	1.881 (0.366)	4.264 (0.587)	2.388 (0.181)	3.634 (0.354)	2.403 (0.078)	3.431 (0.463)	3.217 (0.338)
*Lactobacillus*	17.268 (4.092)	1.956 (0.49)	1.813 (0.351)	1.93 (0.71)	2.49 (0.439)	1.155 (0.165)	6.129 (1.509)
*Bacillus*	1.169 (0.251)	1.379 (0.088)	1.612 (0.079)	1.511 (0.165)	1.476 (0.065)	1.341 (0.055)	1.462 (0.061)
*Roseburia*	0.948 (0.154)	1.621 (0.097)	1.589 (0.042)	1.619 (0.061)	1.517 (0.063)	1.519 (0.112)	1.311 (0.062)
*Butyrivibrio*	0.859 (0.172)	1.56 (0.067)	1.53 (0.036)	1.631 (0.091)	1.563 (0.05)	1.634 (0.083)	1.37 (0.043)
*Ethanoligenens*	0.678 (0.116)	1.235 (0.062)	1.485 (0.021)	1.422 (0.169)	1.578 (0.049)	1.766 (0.171)	1.362 (0.14)
*Holdemania*	0.753 (0.116)	1.305 (0.048)	1.433 (0.058)	1.355 (0.063)	1.379 (0.052)	1.392 (0.035)	1.23 (0.029)
*Streptococcus*	1.019 (0.16)	2.045 (0.553)	1.41 (0.085)	1.226 (0.07)	1.357 (0.123)	1.191 (0.086)	1.224 (0.1)
*Anaerotruncus*	0.655 (0.06)	1.471 (0.102)	1.26 (0.093)	1.777 (0.226)	1.386 (0.159)	1.498 (0.135)	1.094 (0.089)
*Desulfitobacterium*	0.54 (0.135)	1.043 (0.045)	1.052 (0.029)	1.092 (0.06)	1.004 (0.03)	1.111 (0.055)	0.955 (0.031)
*Dorea*	0.894 (0.138)	1.37 (0.058)	1.022 (0.028)	1.384 (0.136)	1.051 (0.043)	1.251 (0.154)	1.147 (0.08)
*Coprococcus*	0.694 (0.097)	1.077 (0.042)	0.9 (0.012)	1.108 (0.089)	0.899 (0.033)	0.992 (0.104)	0.945 (0.056)
*Enterococcus*	1.253 (0.268)	0.843 (0.039)	0.834 (0.02)	0.827 (0.047)	0.803 (0.015)	0.712 (0.046)	0.788 (0.048)
*Escherichia*	7.608 (3.938)	1.296 (0.433)	0.497 (0.191)	0.942 (0.386)	0.686 (0.456)	0.619 (0.225)	1.008 (0.467)
*Eggerthella*	0.436 (0.107)	0.391 (0.015)	0.45 (0.013)	0.516 (0.026)	0.468 (0.019)	0.523 (0.043)	1.072 (0.161)
*Shigella*	1.148 (0.589)	0.203 (0.072)	0.075 (0.03)	0.153 (0.065)	0.105 (0.07)	0.098 (0.036)	0.157 (0.074)
	Crops						
*Faecalibacterium*	1.248 (0.417)	0.487 (0.191)	0.158 (0.066)	0.887 (0.153)	0.092 (0.067)	0.126 (0.015)	0.056 (0.015)
*Ruminococcus*	4.094 (1.287)	2.559 (1.298)	0.334 (0.155)	2.535 (0.366)	0.146 (0.1)	0.495 (0.067)	0.137 (0.048)
*Eubacterium*	2.407 (0.724)	1.389 (0.673)	0.231 (0.095)	1.475 (0.164)	0.126 (0.076)	0.374 (0.031)	0.127 (0.029)
*Bacteroides*	2.294 (0.455)	0.799 (0.427)	0.181 (0.078)	1.149 (0.187)	0.121 (0.08)	0.262 (0.028)	0.092 (0.02)
*Clostridium*	9.578 (2.476)	4.683 (2.122)	0.918 (0.338)	5.655 (0.506)	0.485 (0.311)	1.415 (0.098)	0.483 (0.105)
*Subdoligranulum*	1.597 (0.393)	0.302 (0.095)	0.125 (0.063)	0.539 (0.066)	0.085 (0.076)	0.104 (0.014)	0.051 (0.024)
*Blautia*	1.579 (0.533)	1.15 (0.57)	0.152 (0.082)	0.864 (0.116)	0.053 (0.043)	0.253 (0.022)	0.049 (0.024)
*Lactobacillus*	23.077 (5.819)	59.931 (12.434)	80.761 (6.911)	55.188 (9.781)	81.496 (14.061)	79.209 (5.72)	87.45 (4.139)
*Bacillus*	0.993 (0.129)	0.611 (0.158)	0.576 (0.235)	0.752 (0.212)	0.171 (0.028)	0.551 (0.13)	0.332 (0.08)
*Streptococcus*	0.699 (0.095)	7.536 (4.791)	1.134 (0.562)	1.987 (0.272)	0.612 (0.236)	4.404 (2.505)	1.708 (0.78)
*Enterococcus*	0.628 (0.045)	1.138 (0.446)	0.982 (0.425)	0.641 (0.072)	0.385 (0.103)	1.298 (0.279)	1.05 (0.323)
*Escherichia*	12.382 (6.287)	0.878 (0.404)	0.636 (0.287)	3.344 (1.948)	9.436 (9.311)	2.092 (1.34)	0.97 (0.801)
*Staphylococcus*	0.27 (0.113)	0.134 (0.023)	3.001 (1.338)	0.175 (0.038)	0.276 (0.079)	0.175 (0.035)	1.014 (0.391)
*Corynebacterium*	0.165 (0.117)	0.031 (0.017)	3.648 (2.112)	0.046 (0.024)	0.207 (0.058)	0.011 (0.003)	1.13 (0.79)
*Shigella*	1.858 (0.988)	0.11 (0.054)	0.095 (0.043)	0.491 (0.292)	1.468 (1.446)	0.312 (0.195)	0.15 (0.123)
*Xanthomonas*	1.859 (0.416)	0.87 (0.595)	0.039 (0.023)	2.025 (1.183)	0.043 (0.033)	0.306 (0.047)	0.012 (0.005)
*Klebsiella*	0.137 (0.058)	0.051 (0.024)	0.033 (0.012)	1.016 (0.627)	0.188 (0.171)	0.126 (0.051)	1.161 (1.149)

At 35 days, *Eggerthella* was significantly lower in the caeca of birds treated with the low dose of probiotic in comparison to the control group (0.468 vs 1.072%, p<0.001). The same trend was observed in the caeca of the birds treated with the high dose of probiotic in comparison to the control (0.450 vs 1.072%, p<0.001). Moreover, at the same sampling time (35 days), *Lactobacillus* was significantly lower in the caeca of the high dose group in comparison to the control (1.813 vs 6.129%, p = 0.005). At 14 days, *Enterococcus* and *Streptococcus* were significantly lower in the crops of chickens fed with the low dose of probiotic in comparison to the control (0.641 vs 1.298%, p = 0.006; 1.987 vs 4.404%, p = 0.048). In contrast, *Faecalibacterium* at 14 days was significantly higher in the crops of birds belonging to the low dose group in comparison to the control (0.887 vs 0.126, p = 0.022). *Faecalibacterium* increased also in the high dose group in comparison to the control but that increase was not significantly different. At 35 days, *Corynebacterium* and *Staphylococcus* were significantly higher in the crops of chickens fed the high dose of probiotic in comparison to the low dose group (3.648 vs 0.207%, p = 0.043; 3.001 vs 0.276%, p = 0.0137). Moreover, *Klebsiella* was significantly lower in the crops of birds fed with the high dose of probiotic in comparison to the control (0.033 vs 1.161%, p = 0.011). The covariate results showed that *Bacteroides* significantly decreased in the caeca of birds between 14 and 35 days across all treatments (p = 0.013), while *Eggerthella*, *Faecalibacterium* and *Lactobacillus* significantly increased (p<0.000, p<0.000, and p<0.000). Nevertheless, the abundance of *Eggerthella* was also significantly affected by the interaction between time and treatment (p = 0.041). In the crops, *Lactobacillus* and *Staphylococcus* increased over time (p = 0.007 and p<0.000) while *Xanthomonas* significantly decreased (p = 0.001).

#### Species identified in the caeca and crop microbiome

The species identified in both the caeca and crops of the tested groups across sampling times were *Bacteroides capillosus*, *Clostridium saccharolyticum*, *Escherichia coli*, *Eubacterium rectale*, *Faecalibacterium prausnitzii*, *Lactobacillus acidophilus*, *Lactobacillus crispatus*, *Lactobacillus gasseri*, *Lactobacillus johnsonii*, *Lactobacillus reuteri*, *Ruminococcus torques* and *Subdoligranum variabile* (Table 3). Moreover, specific species were associated with each of the intestinal tracts.

**Table 3:** Species identified in the caeca and crops with a MRA (%) > 1 in at least one treatment (i.e., day 1, high dose (HD) 14 and 35 days, low dose (LD) 14 and 35 days, control (C) 14 and 35 days).

	Mean relative abundance (%) (standard error)						
	Day 1	HD 14d	HD 35d	LD 14d	LD 35d	C 14d	C 35 d
Caeca							
*Anaerotruncus colihominis*	0.628 (0.055)	1.364 (0.095)	1.176 (0.084)	1.652 (0.207)	1.298 (0.147)	1.386 (0.122)	1.025 (0.082)
*Bacteroides capillosus*	3.159 (0.413)	6.444 (0.712)	4.694 (0.178)	5.491 (0.82)	4.265 (0.255)	6.059 (0.806)	4.438 (0.399)
*Blautia hansenii*	1.038 (0.225)	2.713 (0.523)	1.274 (0.14)	1.914 (0.328)	1.219 (0.073)	1.588 (0.234)	1.745 (0.264)
*Blautia hydrogenotrophica*	0.763 (0.133)	1.244 (0.075)	0.959 (0.04)	1.471 (0.095)	1.035 (0.035)	1.592 (0.226)	1.274 (0.088)
*Clostridiales bacterium 1_7_47FAA*	0.593 (0.127)	1.198 (0.131)	0.946 (0.055)	1.221 (0.127)	0.921 (0.048)	1.053 (0.086)	0.765 (0.036)
*Clostridium asparagiforme*	0.743 (0.115)	1.697 (0.223)	1.347 (0.054)	1.663 (0.131)	1.313 (0.07)	1.494 (0.154)	1.056 (0.044)
*Clostridium bolteae*	0.758 (0.107)	1.491 (0.13)	1.211 (0.052)	1.519 (0.129)	1.192 (0.071)	1.278 (0.119)	0.972 (0.037)
*Clostridium botulinum*	0.573 (0.083)	0.94 (0.033)	1.016 (0.022)	1.001 (0.038)	0.962 (0.023)	1.05 (0.056)	0.957 (0.033)
*Clostridium difficile*	0.938 (0.151)	1.738 (0.051)	1.507 (0.026)	1.753 (0.062)	1.578 (0.039)	1.559 (0.067)	1.65 (0.092)
*Clostridium hathewayi*	0.587 (0.079)	1.418 (0.118)	1.136 (0.073)	1.415 (0.154)	1.088 (0.057)	1.22 (0.136)	0.882 (0.044)
*Clostridium leptum*	0.806 (0.129)	1.365 (0.107)	1.226 (0.055)	1.598 (0.168)	1.485 (0.103)	1.797 (0.286)	1.303 (0.108)
*Clostridium nexile*	0.718 (0.134)	1.357 (0.044)	1.06 (0.024)	1.329 (0.1)	1.089 (0.039)	1.152 (0.133)	1.103 (0.074)
*Clostridium phytofermentans*	1.266 (0.227)	2.152 (0.088)	2.048 (0.065)	2.174 (0.09)	1.901 (0.049)	2.099 (0.094)	1.857 (0.072)
*Clostridium proteoclasticum*	0.653 (0.143)	1.2 (0.05)	1.168 (0.02)	1.269 (0.076)	1.227 (0.033)	1.284 (0.064)	1.062 (0.031)
*Clostridium saccharolyticum*	1.692 (0.306)	4.017 (0.411)	3.171 (0.172)	3.803 (0.37)	2.979 (0.106)	3.258 (0.29)	2.465 (0.112)
*Clostridium scindens*	0.704 (0.13)	1.111 (0.032)	0.915 (0.02)	1.13 (0.097)	0.873 (0.043)	1.061 (0.145)	1.03 (0.08)
*Clostridium sp M62 1*	1.065 (0.129)	2.867 (0.836)	1.799 (0.103)	2.166 (0.14)	1.782 (0.11)	1.843 (0.16)	1.437 (0.058)
*Clostridium thermocellum*	0.647 (0.152)	0.994 (0.065)	1.188 (0.036)	1.123 (0.082)	1.141 (0.045)	1.201 (0.097)	1.077 (0.052)
*Desulfitobacterium hafniense*	0.517 (0.127)	0.966 (0.04)	0.983 (0.026)	1.016 (0.054)	0.941 (0.025)	1.028 (0.049)	0.896 (0.028)
*Eggerthella lenta*	0.417 (0.1)	0.362 (0.014)	0.421 (0.012)	0.48 (0.024)	0.439 (0.018)	0.484 (0.039)	1.005 (0.15)
*Escherichia coli*	6.879 (3.556)	1.13 (0.382)	0.436 (0.169)	0.824 (0.342)	0.608 (0.409)	0.535 (0.197)	0.892 (0.418)
*Ethanoligenens harbinense*	0.649 (0.108)	1.144 (0.055)	1.388 (0.02)	1.323 (0.155)	1.48 (0.047)	1.634 (0.156)	1.276 (0.129)
*Eubacterium eligens*	0.809 (0.148)	1.306 (0.045)	1.246 (0.011)	1.295 (0.078)	1.204 (0.026)	1.254 (0.081)	1.176 (0.047)
*Eubacterium rectale*	1.87 (0.286)	2.917 (0.125)	2.574 (0.029)	2.875 (0.148)	2.572 (0.043)	2.762 (0.207)	2.428 (0.108)
*Faecalibacterium prausnitzii*	3.131 (0.877)	1.669 (0.081)	9.199 (1.19)	1.64 (0.06)	8.538 (1.669)	1.998 (0.124)	5.262 (0.664)
*Holdemania filiformis*	0.721 (0.107)	1.209 (0.044)	1.338 (0.051)	1.261 (0.057)	1.292 (0.045)	1.288 (0.03)	1.154 (0.026)
*Lactobacillus acidophilus*	1.801 (0.432)	0.074 (0.011)	0.164 (0.045)	0.086 (0.029)	0.256 (0.065)	0.053 (0.005)	0.827 (0.27)
*Lactobacillus crispatus*	3.887 (1.02)	0.055 (0.006)	0.282 (0.096)	0.112 (0.055)	0.48 (0.139)	0.056 (0.014)	1.661 (0.567)
*Lactobacillus gasseri*	1.201 (0.349)	0.131 (0.048)	0.08 (0.009)	0.151 (0.068)	0.114 (0.023)	0.082 (0.016)	0.227 (0.049)
*Lactobacillus johnsonii*	4.073 (1.061)	0.44 (0.217)	0.164 (0.034)	0.501 (0.307)	0.304 (0.094)	0.227 (0.07)	0.624 (0.152)
*Lactobacillus reuteri*	1.639 (0.477)	0.119 (0.035)	0.189 (0.07)	0.101 (0.034)	0.315 (0.097)	0.056 (0.006)	0.529 (0.16)
*Marvinbryantia formatexigens*	0.871 (0.13)	1.625 (0.085)	1.318 (0.027)	1.508 (0.087)	1.294 (0.031)	1.479 (0.163)	1.351 (0.08)
*uminococcaceae bacterium D16*	1.507 (0.178)	2.536 (0.155)	2.262 (0.07)	2.406 (0.342)	2.112 (0.131)	3.343 (0.466)	2.264 (0.276)
*Ruminococcus albus*	0.925 (0.2)	1.323 (0.044)	1.831 (0.052)	1.539 (0.098)	1.778 (0.061)	1.681 (0.081)	1.626 (0.085)
*Ruminococcus gnavus*	0.83 (0.119)	1.354 (0.068)	0.928 (0.041)	1.485 (0.184)	0.991 (0.058)	1.287 (0.194)	1.101 (0.105)
*Ruminococcus lactaris*	1.05 (0.077)	1.431 (0.143)	0.891 (0.066)	1.74 (0.311)	1.075 (0.106)	1.403 (0.262)	1.133 (0.162)
*Ruminococcus obeum*	0.639 (0.106)	0.968 (0.086)	0.782 (0.039)	1 (0.127)	0.893 (0.04)	0.962 (0.138)	0.98 (0.079)
*Ruminococcus sp 5_1_39BFAA*	0.661 (0.095)	1.034 (0.062)	0.842 (0.028)	1.049 (0.097)	0.856 (0.021)	0.964 (0.124)	0.944 (0.072)
*Ruminococcus torques*	1.298 (0.198)	1.936 (0.242)	1.159 (0.101)	2.317 (0.466)	1.373 (0.159)	1.751 (0.357)	1.493 (0.219)
*Subdoligranulum variabile*	2.356 (0.665)	1.597 (0.133)	2.772 (0.198)	1.408 (0.068)	3.705 (0.455)	4.795 (2.054)	4.298 (1.085)
Crops							
*Bacteroides capillosus*	1.911 (0.393)	0.67 (0.386)	0.124 (0.062)	0.973 (0.151)	0.088 (0.072)	0.219 (0.023)	0.051 (0.02)
*Clostridium saccharolyticum*	1.175 (0.328)	0.625 (0.309)	0.096 (0.047)	0.621 (0.063)	0.046 (0.035)	0.135 (0.023)	0.04 (0.013)
*Escherichia coli*	11.565 (5.959)	0.825 (0.374)	0.597 (0.271)	3.13 (1.835)	8.864 (8.748)	1.982 (1.259)	0.912 (0.754)
*Eubacterium rectale*	1.015 (0.318)	0.627 (0.311)	0.102 (0.045)	0.663 (0.085)	0.052 (0.03)	0.162 (0.016)	0.059 (0.015)
*Faecalibacterium prausnitzii*	1.21 (0.399)	0.477 (0.184)	0.157 (0.066)	0.873 (0.15)	0.092 (0.067)	0.126 (0.015)	0.056 (0.015)
*Klebsiella pneumoniae*	0.131 (0.054)	0.047 (0.021)	0.031 (0.011)	0.914 (0.566)	0.183 (0.167)	0.117 (0.046)	1.107 (1.096)
*Lactobacillus acidophilus*	2.417 (0.58)	14.094 (4.482)	9.331 (1.966)	1.913 (0.297)	7.699 (1.675)	1.741 (0.078)	11.805 (1.49)
*Lactobacillus amylovorus*	0.346 (0.117)	0.778 (0.232)	1.203 (0.171)	0.569 (0.122)	1.046 (0.183)	0.708 (0.115)	1.646 (0.273)
*Lactobacillus crispatus*	5.229 (1.365)	1.739 (0.493)	18.934 (4.468)	2.267 (0.504)	15.572 (3.473)	2.151 (0.369)	21.435 (1.817)
*Lactobacillus fermentum*	0.165 (0.042)	0.45 (0.208)	0.435 (0.175)	0.344 (0.062)	0.619 (0.189)	0.777 (0.223)	1.082 (0.19)
*Lactobacillus gasseri*	1.709 (0.529)	5.159 (1.745)	5.964 (1.372)	7.088 (1.58)	5.843 (1.128)	7.828 (1.365)	4.451 (0.645)
*Lactobacillus helveticus*	1.084 (0.266)	0.982 (0.246)	4.053 (0.781)	0.789 (0.162)	3.479 (0.73)	0.949 (0.119)	6.141 (1.234)
*Lactobacillus johnsonii*	6.757 (2.042)	20.424 (7.671)	29.669 (8.448)	28.993 (6.939)	30.582 (6.877)	36.287 (7.339)	19.769 (4.086)
*Lactobacillus plantarum*	0.172 (0.021)	0.862 (0.532)	0.262 (0.055)	0.575 (0.171)	0.255 (0.061)	1.626 (0.423)	0.877 (0.341)
*Lactobacillus reuteri*	2.158 (0.488)	1.326 (0.45)	4.349 (1.735)	2.872 (1.177)	8.921 (2.385)	1.823 (0.256)	5.861 (1.37)
*Lactobacillus ruminis*	0.028 (0.017)	0.085 (0.032)	0.03 (0.004)	0.05 (0.011)	0.021 (0.003)	1.233 (1.14)	1.048 (0.648)
*Lactobacillus salivarius*	0.328 (0.077)	11.266 (8.419)	1.408 (0.494)	6.862 (2.389)	0.87 (0.446)	20.247 (5.053)	3.467 (1.403)
*Ruminococcus torques*	1.022 (0.34)	0.66 (0.313)	0.075 (0.037)	0.625 (0.135)	0.033 (0.024)	0.118 (0.023)	0.027 (0.009)
*Staphylococcus saprophyticus*	0.041 (0.041)	0.014 (0.009)	1.198 (0.548)	0.019 (0.008)	0.085 (0.029)	0.011 (0.003)	0.354 (0.163)
*Subdoligranulum variabile*	1.551 (0.377)	0.296 (0.091)	0.124 (0.063)	0.53 (0.064)	0.085 (0.076)	0.103 (0.014)	0.051 (0.024)

At 35 days, *Blautia hydrogenotrophica* and *Lactobacillus crispatus* were significantly lower in the caeca of the birds fed the high dose group in comparison to the control (0.959 vs 1.274%, p = 0.035; 0.282 vs 1.661%, p = 0.007). The same trend was observed for *Eggerthella lenta* in both the high dose and low dose groups in comparison to the controls (0.421 vs 1.005%, p<0.000; 0.439 vs 1.005%, p<0.000). On the contrary, at 35 days *Clostridium asparagiforme*, *Clostridium hathewayi* and *Clostridium saccharolyticum* were significantly higher in the caeca of chickens belonging to the high dose group in comparison to the control (1.347 vs 1.056%, p = 0.038; 1.136 vs 0.882%, p = 0.044; 3.171 vs 2.465%, p = 0.044).

At 14 days, *Lactobacillus acidophilus* was significantly higher in the crops of chickens fed with the high dose probiotic in comparison to the control group (14.094 vs 1.741%, p = 0.036). Moreover, the same species was significantly higher in the high dose group in comparison to the low dose group (14.094 vs 1.913%, p<0.000). Other *Lactobacillus* species exhibited an opposite effect. Indeed, *Lactobacillus plantarum* and *Lactobacillus salivarius* were significantly lower at 14 days in the crop of birds fed with low dose of probiotic in comparison to the control (0.575 vs 1.626%, p = 0.019; 6.862 vs 20.247%, p = 0.027). Finally, *Lactobacillus ruminis* was significantly lower in the crop of both low dose and high dose groups at 14 days in comparison to the control (0.05 vs 1.233%, p<0.000; 0.085 vs 1.233%, p = 0.0.36).

At 35 days, *Klebsiella pneumoniae* was significantly lower in the crops of birds fed the low dose of probiotic in comparison to the control (0.183 vs 1.107%, p = 0.006). *Lactobacillus ruminis* displayed the same trend at 35 days in both the low dose (0.021 vs 1.048%, p = 0.001) and high dose groups (0.030 vs 1.048%, p<0.000). Finally, at 35 days *Staphylococcus saprophyticus* was significantly higher in the crops of chickens fed the high dose of probiotic in comparison to those fed the low dose treatment (1.198 vs 0.085%, p = 0.011).

The covariate analysis showed that *Anaerotruncus colihominis*, *Bacteroides capillosus*, *Clostridiales bacterium* 1_7_47FAA, *Clostridium bolteae*, *Clostridium hathewayi*, *Clostridium leptum*, *Clostridium proteoclasticum*, *Clostridium saccharolyticum*, *Desulfitobacterium hafniense* and *Ruminococcaceae bacterium* D16 significantly decreased in the caeca between 14 and 35 days whereas *Clostridium thermocellum*, *Eggerthella lenta*, *Ethanoligenens harbinense*, *Faecalibacterium prausnitzii*, *Lactobacillus acidophilus*, *Lactobacillus crispatus* and *Lactobacillus reuteri* significantly increased over time. Within the crop, *Klebsiella pneumoniae* significantly increased in the control group between 14 and 35 days (0.117 vs 1.107%), whereas within the high and low probiotic groups this species significantly decreased over time (p = 0.006). Different *Lactobacillus* species, including *Lactobacillus acidophilus* (p<0.000) as well as *Lactobacillus amylolyticus* (p<0.000), *Lactobacillus amylovorus* (p<0.000), *Lactobacillus crispatus* p<0.000), *Lactobacillus fermentum* (p = 0.023), *Lactobacillus helveticus* (p<0.000) and *Lactobacillus reuteri* (p<0.000) increased over time in all tested groups. Moreover, at 14 days *Lactobacillus acidophilus* was significantly increased by the high dose treatment (p<0.000). Finally, *Lactobacillus ruminis* significantly decreased in the low dose treatment at at both sampling times (p = 0.025).

### Alpha and beta diversity associated to the genera identified in the caeca and crop microbiome

**Fig 1:**
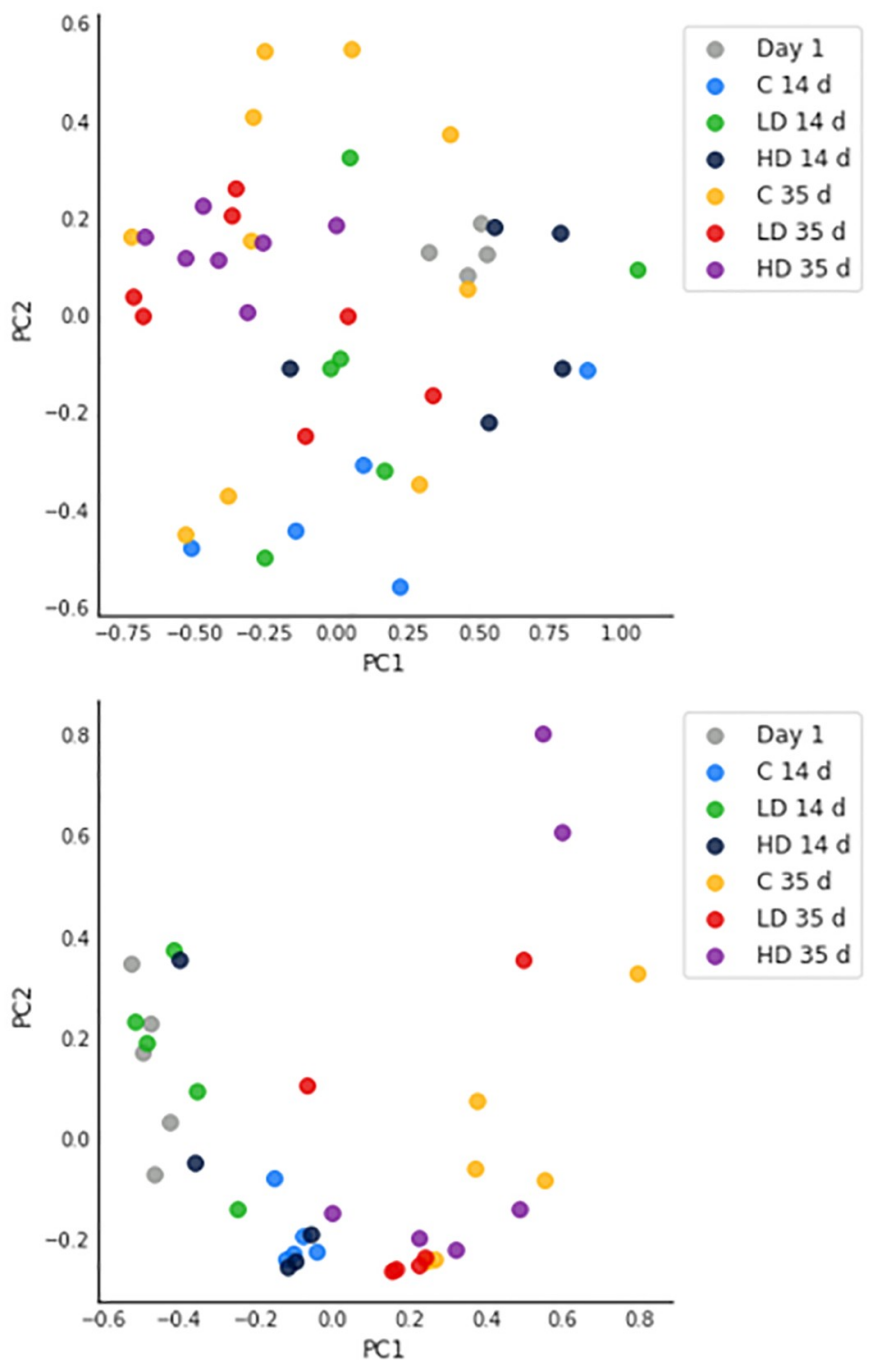
PCoA of beta diversity for the genera identified in the caeca (A) and crops (B) sampled in the control (C), high dose (HD) and low dose (LD) groups at 14 and 35 days.

The alpha diversity values for the genera identified in the caeca and crops of broilers tested in the groups at each sampling time (i.e., 14 and 35 days) were calculated by the Simpson, Shannon and Pielou indexes (S6 Table). The results clearly showed that at the genus level the three indexes of biodiversity calculated for the bacteria colonising the caeca and crops were comparable. The only exception was for the Simpson indexes calculated for the caeca samples which were significantly higher in comparison to the control (p = 0.039) (S7 Table). In relation to the beta diversity, the genera identified in the caeca at day 1, as well as in the high dose group at both 14 and 35 days and in the control group at 14 days clustered separately (Fig 1A), while the genera identified in the low dose group at 14 and 35 days as well as in the control group at 35 days were widely distributed (Fig 1A). In the crops, the genera clustered mainly according to the sampling time. Therefore, the genera identified at day 1 and 14 in all tested groups clustered together separately from the genera identified at 35 days in whatever group (Fig 1B).

### Functional gene composition of caeca and crop microbiome

Table 4 summarizes the functional genes showing a MRA (%) > 0.5 in at least one treatment. The genes decarboxylase, DNA directed RNA polymerase beta subunit EC.2.7.7.6, DNA polymerase III alpha subunit EC.2.7.7.7 and GTP binding protein were detected in both the intestinal tract samples while other genes were associated to a specific region.

**Table 4:** Functional genes with a MRA (%) > 0.5 and corresponding standard error in caeca and crops in at least one treatment (i.e., day 1, high dose (HD) 14 and 35 days, low dose (LD) 14 and 35 days, control (C) 14 and 35 days).

	Mean relative abundance (%) (standard error)						
	Day 1	HD 14d	HD 35d	LD 14d	LD 35d	C 14d	C35 d
Caeca							
Cell division protein FtsH EC.3.4.24	0.576 (0.038)	0.39 (0.016)	0.475 (0.011)	0.383 (0.012)	0.452 (0.013)	0.385 (0.026)	0.428 (0.009)
Copper translocating P type ATPase EC.3.6.3.4.	0.19 (0.033)	0.462 (0.018)	0.407 (0.009)	0.508 (0.022)	0.448 (0.017)	0.489 (0.016)	0.469 (0.014)
Decarboxylase	0.661 (0.167)	0.265 (0.006)	0.25 (0.008)	0.276 (0.014)	0.253 (0.011)	0.28 (0.011)	0.242 (0.008)
DNA directed RNA polymerase beta subunit EC.2.7.7.6.	0.31 (0.075)	0.467 (0.02)	0.576 (0.02)	0.45 (0.015)	0.573 (0.03)	0.439 (0.017)	0.542 (0.023)
DNA polymerase III alpha subunit EC.2.7.7.7.	0.273 (0.05)	0.572 (0.022)	0.752 (0.025)	0.577 (0.011)	0.74 (0.035)	0.576 (0.018)	0.699 (0.03)
DNA topoisomerase III EC.5.99.1.2.	0.164 (0.037)	0.728 (0.096)	0.586 (0.006)	0.747 (0.072)	0.617 (0.024)	0.755 (0.047)	0.563 (0.043)
Excinuclease ABC subunit A	0.352 (0.067)	0.583 (0.035)	0.635 (0.019)	0.643 (0.028)	0.672 (0.014)	0.652 (0.028)	0.695 (0.022)
GTP binding protein	1.057 (0.052)	0.274 (0.005)	0.293 (0.005)	0.28 (0.01)	0.29 (0.009)	0.295 (0.009)	0.304 (0.007)
Retron type reverse transcriptase	0.165 (0.025)	0.431 (0.033)	0.539 (0.022)	0.425 (0.034)	0.404 (0.027)	0.375 (0.058)	0.362 (0.047)
Site specific recombinase	0.188 (0.035)	0.733 (0.019)	0.681 (0.025)	0.815 (0.029)	0.712 (0.034)	0.658 (0.088)	0.518 (0.043)
Crops							
Carbamoyl phosphate synthase large chain EC.6.3.5.5.	0.541 (0.03)	0.495 (0.049)	0.559 (0.038)	0.676 (0.166)	0.475 (0.065)	0.464 (0.069)	0.466 (0.015)
Decarboxylase	0.705 (0.063)	0.624 (0.052)	0.335 (0.019)	0.497 (0.095)	0.285 (0.013)	0.453 (0.014)	0.249 (0.016)
DNA directed RNA polymerase beta subunit EC.2.7.7.6.	0.199 (0.055)	0.342 (0.088)	0.555 (0.031)	0.339 (0.066)	0.508 (0.058)	0.436 (0.051)	0.565 (0.018)
DNA polymerase III alpha subunit EC.2.7.7.7.	0.208 (0.03)	0.415 (0.079)	0.663 (0.012)	0.382 (0.057)	0.576 (0.084)	0.435 (0.047)	0.632 (0.028)
Glycosyltransferase	0.532 (0.047)	0.525 (0.075)	0.427 (0.027)	0.511 (0.017)	0.452 (0.055)	0.527 (0.049)	0.437 (0.027)
GTP binding protein	1.491 (0.179)	1.059 (0.251)	0.334 (0.025)	1.054 (0.181)	0.358 (0.034)	0.716 (0.026)	0.309 (0.011)
Oligopeptide ABC transporter periplasmic oligopeptide binding protein OppA TC.3.A.1.5.1.	0.087 (0.029)	0.511 (0.109)	0.766 (0.071)	0.372 (0.078)	0.639 (0.093)	0.606 (0.067)	0.785 (0.059)
X6 phospho beta glucosidase EC.3.2.1.86.	0.153 (0.037)	0.403 (0.097)	0.512 (0.073)	0.349 (0.096)	0.51 (0.081)	0.589 (0.043)	0.484 (0.053)

At 35 days, the Cell division protein FtsH (EC 3.4.24.-) gene significantly increased in the caeca of birds treated with the high dose of probiotic in comparison to the control group (0.475 vs 0.428%, p = 0.016). Moreover, the Site-specific recombinase gene significantly increased in both the low dose and high dose groups in comparison to the control (0.712 vs 0.518%, p = 0.007; 0.681 vs 0.518%, p = 0.017). In contrast, the Copper-translocating P-type ATPase (EC 3.6.3.4) gene significantly decreased at 35 days in the caeca of birds treated with the high dose of probiotic in comparison to the control (0.407 vs 0.469%, p = 0.044). The covariate analysis showed that in the caeca the DNA polymerase III alpha subunit (EC 2.7.7.7) gene significantly increased between 14 and 35 days (p = 0.005), whereas DNA topoisomerase III (EC 5.99.1.2) significantly decreased. Within the crop, the decarboxylase gene significantly decreased over time (p = 0.005) whereas DNA polymerase III alpha subunit (EC 2.7.7.7) and Oligopeptide ABC transporter, periplasmic oligopeptide-binding protein OppA (TC 3.A.1.5.1) significantly increased (p = 0.024 and p = 0.032).

## Discussion

In this research we deeply explored the effects of supplementing LA in broiler drinking water on the caeca and crop microbiome in terms of taxonomic and functional gene composition without considering the impact of the treatment on animal performance parameters. However, besides the probiotic administration strategy and the animal numerousness, the birds investigated in this study were reared as described in a previous study where we assessed the effects of the supplementation of the same probiotic in the chicken feed on productive performances and foot pad dermatitis.

The results showed that the MRA of *Lactobacillus acidophilus* in the caeca did not show significative differences between the treated and control birds, although *Lactobacillus acidophilus* as well as other microorganisms promoting the chicken’s health, including *Faecalibacterium prausnitzii*, *Lactobacillus crispatus* and *Lactobacillus reuteri*, significantly increased over time. Conversely, the administration of the high dose probiotic significantly affected the abundance of *Lactobacillus acidophilus* in the chicken crops, at least in the first rearing period, demonstrating that the crop is the main site where the effects of the probiotic initially take place. Indeed, at 14 days *Lactobacillus acidophilus* was significantly higher in the crops of chickens treated with the high dose of LA in comparison to the control (14.094 vs 1.741%, p = 0.036). At 35 days this difference disappeared but there was an overall increase of other *Lactobacillus* species, including *Lactobacillus amylolyticus*, *Lactobacillus amylovorus*, *Lactobacillus crispatus*, *Lactobacillus fermentum*, *Lactobacillus helveticus* and *Lactobacillus reuteri* which might in some way compete with *Lactobacillus acidophilus* thus affecting its abundance. A further explanation might be found in the covariate analysis results (treatment, sampling time and their interactions) since the increase of the microbial diversity over time may decrease the ability to detect treatment effects on the abundance of *Lactobacillus acidophilus* as well as other species. Therefore, the treatment effect may only be detectable within each separate sampling time.

In the crop, the effect of the different concentrations of probiotic showed up and the better results were obtained with the low dose probiotic treatment which significantly increased the abundance of the genus *Faecalibacterium* compared to the control while no difference was observed between the high dose and the control group. *Faecalibacterium* is one of the most abundant butyrate producers in the hindgut of both humans and other monogastric animals [24, 25]. It plays a key role in maintaining gut health as the major source of energy to the colonic mucosa and is an important regulator of gene expression, inflammation, differentiation, and apoptosis in host cells [26–28]. One hypothesis to the better effect of the LA supplemented in the low dose in comparison to the high dose on the improvement of the beneficial microbes in the broiler gastrointestinal tract might be the hormesis, a dose–response phenomenon in which opposite effects are observed at low, compared to high doses for the same measured parameter [25]. However, this phenomenon has been never described for lactic acid bacteria.

Within the caeca, a positive high dose treatment effect was the significant increase in the abundance of *Clostridium asparagiforme*, *Clostridium hathewayi* and *Clostridium saccharolyticum* in comparison to the control group at 35 days. *Clostridium saccharolyticum* can utilize carbohydrates or polysaccharides as carbon sources and produce acetate, propionate and butyrate as fermented products [29]. Moreover, *Clostridium hathewayi* produce acetate and *Clostridium asparagiforme* lactate [30].

At 35 day, the decrease of Actinobacteria in the caeca of chickens belonging to the low and high dose groups in comparison to the control group corresponded to the decrease of the genus *Corynebacterium* and the species *Eggerthella lenta*. This species has been described by Haiser et al., 2013 [31] as part of the human gut microbiome. The same study showed that the fastidious growth of *Eggerthella lenta* is promoted by growth factors supplied by the gut microbiota and its abundance increased in the presence of a complex microbial community. In our study the overall microbial complexity, calculated at the genus level, was quite similar in the caeca of chickens tested at 35 days in all groups, although the Simpson index calculated for the caeca of the birds treated with the probiotic was significantly higher than the control group, which translates to a less diverse microbial composition in the caeca of treated birds in comparison to the control. Although this trend was detected using the Simpson index only, our hypothesis to explain the decrease of *Eggerthella lenta* in the caeca of the treated animals is the overall decrease of the microbial biodiversity in those groups. A second hypothesis might be that the supplemented *Lactobacillus acidophilus* competes with *Eggerthella lenta* for arginine. Indeed, Trinchieri et al., 2011 [27] showed that some lactic acid bacteria are rich in arginine deiminase which catalyses the irreversible conversion of arginine to citrulline and ammonia. This conversion decreases the availability of arginine, which is an important energy source for *Eggerthella lenta* that possesses genes for arginine deiminase (arcA), ornithine carbamoyltransferase (argF or agI), and carbamate kinase (arcC) [32].

In relation to the beta diversity, the genera identified in the caeca at day 1 (Fig 1A) clustered all together. This finding was expected because the intestinal microbiota richness, i.e., the number of different microbial taxa, increases over time [33–35]. More interestingly, the genera identified in the high dose groups at both 14 and 35 days clustered separately. This result demonstrates that beside the variations in the abundance of single taxonomic groups, the overall caeca population was affected by the administration of LA at the highest dose in terms of composition. However, this effect was not observed in the crops, where the genera clustered mainly according to the sampling time (Fig 1B).

The most abundant functional genes were different in between the crop and caeca. At 35 days, the Cell division protein FtsH (EC 3.4.24.-) and the Site-specific recombinase gene were significantly increased in the caeca of birds treated with the high dose of probiotic in comparison to the control group. The cell division protein FtsH is a peptidase essential in bacterial stress response, including *Lactococcus lactis* and *Bacillus subtilis* [36]. Therefore, its increase in the caeca of birds fed with the high dose of probiotic highlights an overall beneficial effect of the probiotic on the microorganisms colonizing the gut of treated animals. The increase in abundance of the site-specific recombinase gene is more difficult to explain, although it might be related to some form of stress response because the site-specific recombinase gene in prokaryotes is part of the inversional switching systems along with the invertible DNA segments and they mediate alternative expression of sets of genes [37]. Besides these potential positive effects of the high dose probiotic treatment on the caeca microbiome, there was a negative effect related to the decrease of the Copper-translocating P-type ATPase (EC 3.6.3.4) gene. This gene encodes for one of the proteins required for the transport and delivery of copper, an essential cellular component which is required for a broad range of enzymes involved in numerous metabolic pathways, including respiration and free radical scavenging [38]. Only trace amounts of copper are needed to sustain life and the decrease of the Copper-translocating P-type ATPase gene in the caeca of treated animals might result in an excess of copper in the cells which is extremely toxic for their viability.

## Conclusions

The results of this study showed that *Lactobacillus acidophilus* D2/CSL (CECT 4529) supplementation in the drinking water at the concentrations of 0.2 and 0.02 g *Lactobacillus acidophilus*/day/bird improved beneficial microbes and functional genes in broiler crops and caeca although the main site of action of the probiotic is the crop. In the crop, the better effect on the beneficial microbes was obtained supplementing the probiotic in the drinking water at the lower dose.

## Supporting information

S1 Table Labels of the metagenomes investigated in each tested group at the different sampling time (day 1, day 14, day 35). All the metagenomes are publicly available in MG-RAST.
(DOCX)

S2 Table Phyla identified in the caeca and crops with a MRA (%) > 1 in at least one treatment (day 1, high dose (HD) 14 and 35 days (d), low dose (LD) 14 and 35 days, control (C) 14 and 35 days). (DOCX)

S3 Table Classes identified in the caeca and crops with a MRA (%) > 1 in at least one treatment (i.e., day 1, high dose (HD) 14 and 35 days, low dose (LD) 14 and 35 days, control (C) 14 and 35 days). (DOCX)

S4 Table Orders identified in the caeca and crops with a MRA (%) > 1 in at least one treatment (i.e., day 1, high dose (HD) 14 and 35 days, low dose (LD) 14 and 35 days, control (C) 14 and 35 days). (DOCX)

S5 Table Families identified in the caeca and crops with a MRA (%) > 1 in at least one treatment (i.e., day 1, high dose (HD) 14 and 35 days, low dose (LD) 14 and 35 days, control (C) 14 and 35 days). (DOCX)

S6 Table Mean values of the Simpson, Shannon and Pielou indexes quantified for the genera identified in the caeca and crops of chickens belonging to the tested treatments (i.e., day 1, high dose (HD) 14 and 35 days, low dose (LD) 14 and 35 days, control (C) 14 and 35 days). (DOCX)

S7 Table P values calculated for the Simpson, Shannon and Pielou indexes quantified for the genera identified in the caeca and crops of chickens belonging to the tested treatments (i.e., day 1, high dose (HD) 14 and 35 days, low dose (LD) 14 and 35 days, control (C) 14 and 35 days). (DOCX)
