# Supplementary material for: Effect of Lactobacillus acidophilus D2/CSL (CECT 4529) supplementation in drinking water on chicken crop and caeca microbiome
Source: PLoS One. 2020 Jan 24;15(1):e0228338. doi: 10.1371/journal.pone.0228338 (PMC6980619; doi:10.1371/journal.pone.0228338)
Supplement: S1 Table — All the metagenomes are publicly available in MG-RAST. (DOCX) [file pone.0228338.s001.docx]

**S1 Table. Labels of the metagenomes investigated in each tested group at the different sampling time (day 1, day 14, day 35). All the metagenomes are publicly available in MG-RAST**

| **Caeca day 1** |
| --- |
| mgm4624898.3 |
| mgm4625263.3 |
| mgm4625261.3 |
| mgm4625265.3 |
| **Crop day 1** |
| mgm4624894.3 |
| mgm4624891.3 |
| mgm4624893.3 |
| mgm4624887.3 |
| mgm4624875.3 |
| **Caeca day 14 low dose** |
| mgm4625281.3 |
| mgm4624901.3 |
| mgm4624880.3 |
| mgm4624878.3 |
| mgm4624873.3 |
| mgm4624881.3 |
| **Caeca day 14 high dose** |
| mgm4624876.3 |
| mgm4624885.3 |
| mgm4624890.3 |
| mgm4624892.3 |
| mgm4624902.3 |
| **Caeca day 14 control** |
| mgm4625295.3 |
| mgm4625302.3 |
| mgm4625271.3 |
| mgm4625310.3 |
| mgm4625278.3 |
| **Crops day 14 low dose** |
| mgm4625294.3 |
| mgm4625296.3 |
| mgm4625303.3 |
| mgm4625299.3 |
| mgm4625314.3 |
| **Crops day 14 high dose** |
| mgm4625284.3 |
| mgm4625264.3 |
| mgm4625298.3 |
| mgm4625301.3 |
| mgm4625268.3 |
| **Crops day 14 control** |
| mgm4625291.3 |
| mgm4625306.3 |
| mgm4625289.3 |
| mgm4625305.3 |
| mgm4625292.3 |
| **Caeca day 35 low dose** |
| mgm4624882.3 |
| mgm4624895.3 |
| mgm4624879.3 |
| mgm4624889.3 |
| mgm4624900.3 |
| mgm4624888.3 |
| mgm4624899.3 |
| **Caeca day 35 high dose** |
| mgm4624897.3 |
| mgm4624874.3 |
| mgm4624886.3 |
| mgm4624896.3 |
| mgm4624877.3 |
| mgm4624883.3 |
| mgm4624884.3 |
| **Caeca day 35 control** |
| mgm4625276.3 |
| mgm4625309.3 |
| mgm4625321.3 |
| mgm4625293.3 |
| mgm4625275.3 |
| mgm4625307.3 |
| mgm4625311.3 |
| mgm4625279.3 |
| mgm4625274.3 |
| mgm4625270.3 |
| **Crops day 35 low dose** |
| mgm4625317.3 |
| mgm4625283.3 |
| mgm4625318.3 |
| mgm4625322.3 |
| mgm4625319.3 |
| mgm4625313.3 |
| **Crops day 35 high dose** |
| mgm4625280.3 |
| mgm4625286.3 |
| mgm4625312.3 |
| mgm4625300.3 |
| mgm4625266.3 |
| mgm4625282.3 |
| **Crops day 35 control** |
| mgm4625308.3 |
| mgm4625315.3 |
| mgm4625320.3 |
| mgm4625277.3 |
| mgm4625267.3 |
| mgm4625290.3 |
