# Supplementary material for: Effect of Lactobacillus acidophilus D2/CSL (CECT 4529) supplementation in drinking water on chicken crop and caeca microbiome
Source: PLoS One. 2020 Jan 24;15(1):e0228338. doi: 10.1371/journal.pone.0228338 (PMC6980619; doi:10.1371/journal.pone.0228338)
Supplement: S2 Table — (DOCX) [file pone.0228338.s002.docx]

**S2 Table. Phyla identified in the caeca and crops with a MRA (%) > 1 in at least one treatment (day 1, high dose (HD) 14 and 35 days (d), low dose (LD) 14 and 35 days, control (C) 14 and 35 days).**

|  | Mean relative abundance (%) (standard error) in the caeca | | | | | | | | |
| --- | --- | --- | --- | --- | --- | --- | --- | --- | --- |
| Phylum | Day 1 | HD 14 d | HD 35 d | LD 14 d | LD 35 d | | C 14 d | | C 35 d |
| Caeca | | | | | | | | | |
| Firmicutes | 68.815(4.977) | 81.861 (0.827) | 83.262 (0.236) | 82.479 (0.841) | 83.540 (0.366) | | 82.017(1.255) | | 82.118 (0.975) |
| Bacteroidetes | 4.261  (0.532) | 7.783 (0.730) | 6.136 (0.200) | 6.910 (0.804) | 5.713  (0.285) | | 7.446 (0.800) | | 5.756  (0.386) |
| Proteobacteria | 20.881 (6.037) | 4.531 (0.445) | 3.955 (0.216) | 4.258 (0.373) | 4.172 (0.533) | | 3.985 (0.300) | | 4.495 (0.566) |
| Actinobacteria | 2.866 (0.543) | 2.667 (0.068) | 3.012 (0.033) | 2.953 (0.095) | 3.046 (0.045) | | 3.108 (0.150) | | 4.189 (0.362) |
| Crops | | | | | | | | | |
| Firmicutes | 56.608 (6.485) | 85.603 (6.659) | 90.353 (4.198) | 77.768 (7.733) | 84.951 (12.671) | 91.932 (2.231) | | 94.169 (2.767) | |
| Bacteroidetes | 2.805 (0.377) | 1.188  (0.654) | 0.255 (0.089) | 1.578 (0.330) | 0.266  (0.130) | 0.405 (0.030) | | 0.157 (0.024) | |
| Proteobacteria | 35.406 (7.317) | 10.674 (5.074) | 1.964 (0.479) | 17.155 (6.342) | 13.945 (12.352) | 6.673 (2.150) | | 3.221 (2.430) | |
| Actinobacteria | 1.779 (0.354) | 0.695 (0.200) | 7.150 (3.753) | 1.217  (0.247) | 0.653  (0.171) | 0.319 (0.027) | | 2.281 (1.291) | |
