# Supplementary material for: Effect of Lactobacillus acidophilus D2/CSL (CECT 4529) supplementation in drinking water on chicken crop and caeca microbiome
Source: PLoS One. 2020 Jan 24;15(1):e0228338. doi: 10.1371/journal.pone.0228338 (PMC6980619; doi:10.1371/journal.pone.0228338)
Supplement: S3 Table — (DOCX) [file pone.0228338.s003.docx]

**S3 Table. Classes identified in the caeca and crops** **with a MRA (%) > 1 in at least one treatment (i.e., day 1, high dose (HD) 14 and 35 days, low dose (LD) 14 and 35 days, control (C) 14 and 35 days).**

|  | | Mean relative abundance (%) (standard error) | | | | | | | | | | |  |
| --- | --- | --- | --- | --- | --- | --- | --- | --- | --- | --- | --- | --- | --- |
| Class | | Day 1 | HD 14 d | | HD 35 d | LD 14 d | | LD 35 d | C 14 d | | C 35 d | |  |
| Caeca | | | | | | | | | | | | |  |
| Clostridia | | 43.359 (7.012) | 70.067 (1.807) | | 71.327 (0.703) | 71.335 (1.013) | | 71.569 (0.679) | 72.322 (1.312) | | 66.883 (1.968) | |  |
| Bacilli | | 22.329(3.865) | 7.958 (0.993) | | 7.875 (0.450) | 7.431 (0.948) | | 8.160 (0.587) | 6.270 (0.177) | | 11.591 (1.599) | |  |
| Bacteroidia | | 4.115 (0.513) | 7.572 (0.727) | | 5.890 (0.196) | 6.675 (0.805) | | 5.474 (0.278) | 7.213 (0.794) | | 5.524 (0.388) | |  |
| Erysipelotrichi | | 1.763 (0.287) | 3.256 (0.186) | | 3.493  (0.208) | 3.078 (0.173) | | 3.245 (0.234) | 2.768  (0.073) | | 3.092 (0.163) | |  |
| Actinobacteria | | 2.885 (0.545) | 2.676 (0.069) | | 3.025 (0.033) | 2.965 (0.096) | | 3.059 (0.045) | 3.121 (0.152) | | 4.206 (0.364) | |  |
| Gammaproteobacteria | | 19.139 (6.099) | 2.700 (0.543) | | 1.837 (0.227) | 2.294 (0.438) | | 2.064 (0.555) | 1.922 (0.271) | | 2.424 (0.579) | |  |
| Negativicutes | | 1.837 (0.169) | 0.870 (0.029) | | 0.977 (0.014) | 0.951 (0.031) | | 0.919  (0.014) | 0.989  (0.038) | | 0.886 (0.030) | |  |
| Crops | | | | | | | | | | | | | |
| Clostridia | 27.046 (7.008) | | | 13.677  (6.244) | 2.606 (1.024) | | 16.079 (1.611) | 1.338 (0.907) | | 3.769 (0.262) | | 1.275  (0.310) | |
| Bacilli | 26.494 (5.620) | | | 70.466 (12.149) | 87.580 (4.385) | | 59.689 (9.101) | 83.486 (13.674) | | 87.618 (2.374) | | 92.801 (3.016) | |
| Bacteroidia | 2.764 (0.371) | | | 1.108 (0.621) | 0.202 (0.074) | | 1.512 (0.333) | 0.144 (0.094) | | 0.357 (0.027) | | 0.109 (0.020) | |
| Erysipelotrichi | 1.173 (0.276) | | | 0.672 (0.325) | 0.131 (0.045) | | 0.648 (0.074) | 0.068 (0.041) | | 0.190 (0.019) | | 0.075 (0.015) | |
| Actinobacteria | 1.797 (0.356) | | | 0.700 (0.203) | 7.154 (3.756) | | 1.233 (0.257) | 0.653 (0.172) | | 0.319 (0.027) | | 2.282 (1.292) | |
| Gammaproteobacteria | 33.253 (7.319) | | | 9.379 (4.436) | 1.576 (0.403) | | 15.678 (5.926) | 13.039 (12.409) | | 5.943 (2.098) | | 2.944 (2.395) | |
| Negativicutes | 2.468 (0.157) | | | 1.152 (0.658) | 0.080 (0.035) | | 1.936 (0.138) | 0.081 (0.061) | | 0.527 (0.085) | | 0.049 (0.009) | |
