# Supplementary material for: Effect of Lactobacillus acidophilus D2/CSL (CECT 4529) supplementation in drinking water on chicken crop and caeca microbiome
Source: PLoS One. 2020 Jan 24;15(1):e0228338. doi: 10.1371/journal.pone.0228338 (PMC6980619; doi:10.1371/journal.pone.0228338)
Supplement: S4 Table — (DOCX) [file pone.0228338.s004.docx]

**S4 Table. Orders identified in the caeca and crops with a MRA (%) > 1 in at least one treatment (i.e., day 1, high dose (HD) 14 and 35 days, low dose (LD) 14 and 35 days, control (C) 14 and 35 days).**

| Mean relative abundance (%) | | | | | | | |
| --- | --- | --- | --- | --- | --- | --- | --- |
|  | Day 1 | HD 14d | HD 35d | LD 14d | LD 35d | C 14d | C35 d |
| Caeca | | | | | | | |
| Clostridiales | 43.358 (6.945) | 68.327 (1.863) | 69.224 (0.733) | 69.414 (1.102) | 69.538 (0.725) | 70.249 (1.483) | 64.95 (1.94) |
| Bacteroidales | 4.206 (0.529) | 7.569 (0.726) | 5.887 (0.196) | 6.674 (0.805) | 5.471 (0.278) | 7.211 (0.792) | 5.522 (0.387) |
| Lactobacillales | 20.04 (4.416) | 5.05 (0.894) | 4.397 (0.312) | 4.262 (0.722) | 4.935 (0.485) | 3.339 (0.165) | 8.273 (1.528) |
| Bacillales | 2.832 (0.531) | 2.906 (0.167) | 3.474 (0.149) | 3.168 (0.27) | 3.221 (0.137) | 2.93 (0.14) | 3.315 (0.132) |
| Erysipelotrichales | 1.802 (0.298) | 3.255 (0.186) | 3.438 (0.208) | 3.077 (0.173) | 3.244 (0.234) | 2.767 (0.073) | 3.091 (0.163) |
| Selenomonadales | 1.881 (0.186) | 0.87 (0.029) | 0.976 (0.014) | 0.951 (0.031) | 0.919 (0.014) | 0.989 (0.038) | 0.886 (0.03) |
| Enterobacteriales | 14.76 (6.162) | 1.866 (0.551) | 0.88 (0.231) | 1.407 (0.465) | 1.117 (0.561) | 1.028 (0.27) | 1.502 (0.582) |
| Actinomycetales | 1.335 (0.421) | 0.691 (0.029) | 0.806 (0.015) | 0.75 (0.032) | 0.807 (0.019) | 0.795 (0.04) | 1.366 (0.339) |
| Coriobacteriales | 1.152 (0.186) | 1.269 (0.036) | 1.45 (0.018) | 1.417 (0.056) | 1.449 (0.038) | 1.528 (0.104) | 2.087 (0.212) |
| Xanthomonadales | 1.102 (0.273) | 0.035 (0.002) | 0.044 (0.001) | 0.039 (0.002) | 0.045 (0.001) | 0.041 (0.004) | 0.041 (0.003) |
| Crops | | | | | | | |
| Clostridiales | 28.316 (7.6) | 13.943 (6.398) | 2.532 (1.012) | 16.064 (1.675) | 1.302 (0.887) | 3.707 (0.265) | 1.222 (0.304) |
| Bacteroidales | 2.929 (0.42) | 1.16 (0.668) | 0.203 (0.084) | 1.538 (0.342) | 0.144 (0.094) | 0.36 (0.027) | 0.109 (0.02) |
| Lactobacillales | 26.072 (6.003) | 70.533 (12.31) | 83.543 (6.004) | 58.992 (9.352) | 82.85 (13.781) | 87.126 (2.57) | 91.08 (3.325) |
| Bacillales | 1.849 (0.244) | 1.2 (0.249) | 4.118 (1.725) | 1.47 (0.271) | 0.629 (0.119) | 1.216 (0.285) | 1.713 (0.511) |
| Erysipelotrichales | 1.253 (0.308) | 0.697 (0.345) | 0.131 (0.046) | 0.658 (0.076) | 0.068 (0.041) | 0.191 (0.019) | 0.075 (0.015) |
| Selenomonadales | 2.608 (0.177) | 1.211 (0.71) | 0.08 (0.036) | 1.97 (1.16) | 0.081 (0.06) | 0.531 (0.084) | 0.049 (0.009) |
| Enterobacteriales | 24.741 (7.254) | 5.374 (2.393) | 1.148 (0.342) | 9.464 (3.663) | 12.623 (12.225) | 4.359 (1.964) | 2.699 (2.373) |
| Actinomycetales | 0.915 (0.299) | 0.291 (0.063) | 6.98 (3.733) | 0.533 (0.14) | 0.508 (0.129) | 0.149 (0.026) | 2.113 (1.287) |
| Coriobacteriales | 0.638 (0.147) | 0.284 (0.123) | 0.059 (0.021) | 0.547 (0.124) | 0.042 (0.023) | 0.099 (0.018) | 0.044 (0.005) |
| Xanthomonadales | 2.451 (0.563) | 1.02 (0.679) | 0.053 (0.027) | 2.752 (1.479) | 0.067 (0.044) | 0.36 (0.051) | 0.019 (0.008) |
