# Supplementary material for: Effect of Lactobacillus acidophilus D2/CSL (CECT 4529) supplementation in drinking water on chicken crop and caeca microbiome
Source: PLoS One. 2020 Jan 24;15(1):e0228338. doi: 10.1371/journal.pone.0228338 (PMC6980619; doi:10.1371/journal.pone.0228338)
Supplement: S5 Table — (DOCX) [file pone.0228338.s005.docx]

**S5 Table. Families identified in the caeca and crops with a MRA (%) > 1 in at least one treatment (i.e., day 1, high dose (HD) 14 and 35 days, low dose (LD) 14 and 35 days, control (C) 14 and 35 days).**

|  | Mean relative abundance (%) (standard error) | | | | | | |
| --- | --- | --- | --- | --- | --- | --- | --- |
|  | Day 1 | HD  14 d | HD  35 d | LD  14 d | LD  35 d | C  14 d | C  35 d |
| Caeca | | | | | | | |
| Lactobacillaceae | 17.89 (4.332) | 1.983 (0.484) | 1.831 (0.34) | 1.963 (0.705) | 2.503 (0.431) | 1.179 (0.163) | 6.138 (1.491) |
| Clostridiaceae | 15.836 (2.622) | 30.464 (1.86) | 26.45 (0.649) | 30.138 (0.961) | 25.974 (0.771) | 28.189 (1.625) | 24.168 (0.739) |
| Ruminococcaceae | 14.915 (2.604) | 17.851 (0.48) | 24.799 (1.341) | 19.109 (0.988) | 25.698 (1.664) | 22.891 (2.181) | 22.957 (1.74) |
| Enterobacteriaceae | 15.168 (6.288) | 1.978 (0.58) | 0.918 (0.243) | 1.487 (0.49) | 1.161 (0.582) | 1.08 (0.284) | 1.563 (0.604) |
| Lachnospiraceae | 5.023 (0.762) | 8.381 (0.35) | 7.353 (0.132) | 8.435 (0.474) | 7.313 (0.228) | 7.877 (0.645) | 7.122 (0.343) |
| Eubacteriaceae | 4.779 (0.89) | 7.258 (0.21) | 6.663 (0.074) | 7.42 (0.286) | 6.724 (0.103) | 7.115 (0.384) | 6.602 (0.273) |
| Bacteroidaceae | 3.874 (0.532) | 7.703 (0.732) | 5.771 (0.198) | 6.695 (0.826) | 5.328 (0.284) | 7.237 (0.797) | 5.447 (0.391) |
| Streptococcaceae | 1.132 (0.17) | 2.113 (0.549) | 1.499 (0.086) | 1.299 (0.074) | 1.431 (0.123) | 1.253 (0.088) | 1.319 (0.106) |
| Erysipelotrichaceae | 1.868 (0.316) | 3.449 (0.18) | 3.576 (0.214) | 3.246 (0.18) | 3.374 (0.244) | 2.91 (0.088) | 3.238 (0.177) |
| Bacillaceae | 1.544 (0.359) | 1.835 (0.117) | 2.184 (0.107) | 2.022 (0.223) | 1.993 (0.09) | 1.776 (0.074) | 2.009 (0.088) |
| Coriobacteriaceae | 1.194 (0.198) | 1.346 (0.036) | 1.509 (0.019) | 1.494 (0.055) | 1.507 (0.039) | 1.605 (0.103) | 2.180 (0.218) |
| Peptococcaceae | 0.894 (0.239) | 1.644 (0.069) | 1.743 (0.043) | 1.734 (0.09) | 1.685 (0.048) | 1.77 (0.085) | 1.604 (0.051) |
| Enterococcaceae | 1.285 (0.285) | 0.832 (0.035) | 0.814 (0.018) | 0.816 (0.048) | 0.787 (0.015) | 0.697 (0.048) | 0.778 (0.05) |
| Veillonellaceae | 1.632 (0.222) | 0.514 (0.013) | 0.565 (0.014) | 0.556 (0.017) | 0.524 (0.021) | 0.553 (0.017) | 0.523 (0.013) |
| Xanthomonadaceae | 1.137 (0.277) | 0.037 (0.002) | 0.046 (0.001) | 0.041 (0.003) | 0.046 (0.001) | 0.043 (0.004) | 0.043 (0.003) |
| Thermoanaerobacteraceae | 0.436 (0.123) | 0.883 (0.048) | 1.059 (0.029) | 0.991 (0.064) | 1.015 (0.035) | 1.074 (0.081) | 0.989 (0.044) |
| Crops | | | | | | | |
| Lactobacillaceae | 25.06 (6.147) | 61.882 (12.572) | 81.096 (6.922) | 56.940 (9.714) | 81.682 (14.086) | 81.024 (5.526) | 87.911 (4.165) |
| Clostridiaceae | 10.752 (2.892) | 5.064 (2.297) | 0.958 (0.349) | 6.012 (0.553) | 0.501 (0.319) | 1.473 (0.103) | 0.498 (0.107) |
| Ruminococcaceae | 9.342 (2.575) | 4.088 (1.927) | 0.727 (0.329) | 5.076 (0.565) | 0.395 (0.302) | 0.934 (0.101) | 0.291 (0.102) |
| Enterobacteriaceae | 25.397 (7.312) | 5.546 (2.527) | 1.151 (0.343) | 9.736 (3.82) | 12.692 (12.294) | 4.379 (1.973) | 2.705 (2.379) |
| Lachnospiraceae | 3.544 (0.826) | 1.947 (0.928) | 0.298 (0.127) | 2.047 (0.289) | 0.139 (0.106) | 0.434 (0.058) | 0.132 (0.034) |
| Eubacteriaceae | 2.671 (0.836) | 1.477 (0.72) | 0.237 (0.098) | 1.544 (0.169) | 0.129 (0.078) | 0.38 (0.032) | 0.13 (0.029) |
| Bacteroidaceae | 2.487 (0.524) | 0.856 (0.474) | 0.182 (0.079) | 1.193 (0.209) | 0.121 (0.08) | 0.265 (0.028) | 0.093 (0.02) |
| Streptococcaceae | 0.817 (0.129) | 8.097 (4.927) | 1.216 (0.576) | 2.166 (0.303) | 0.677 (0.229) | 4.707 (2.627) | 1.832 (0.789) |
| Erysipelotrichaceae | 1.303 (0.331) | 0.725 (0.362) | 0.131 (0.046) | 0.673 (0.078) | 0.068 (0.041) | 0.192 (0.02) | 0.075 (0.015) |
| Bacillaceae | 1.17 (0.177) | 0.729 (0.182) | 0.77 (0.323) | 0.894 (0.226) | 0.211 (0.033) | 0.69 (0.16) | 0.453 (0.113) |
| Corynebacteriaceae | 0.174 (0.121) | 0.033 (0.019) | 3.653 (2.115) | 0.046 (0.025) | 0.207 (0.058) | 0.011 (0.003) | 1.131 (0.79) |
| Enterococcaceae | 0.672 (0.033) | 1.173 (0.455) | 0.985 (0.426) | 0.663 (0.08) | 0.385 (0.103) | 1.313 (0.283) | 1.051 (0.324) |
| Veillonellaceae | 2.54 (0.2) | 1.206 (0.734) | 0.066 (0.031) | 1.919 (1.197) | 0.073 (0.056) | 0.503 (0.081) | 0.038 (0.007) |
| Xanthomonadaceae | 2.519 (0.567) | 1.061 (0.715) | 0.053 (0.027) | 2.837 (1.54) | 0.067 (0.044) | 0.362 (0.051) | 0.019 (0.008) |
| Staphylococcaceae | 0.294 (0.119) | 0.155 (0.025) | 3.118 (1.382) | 0.194 (0.042) | 0.288 (0.081) | 0.182 (0.039) | 1.039 (0.4) |
