# Supplementary material for: Effect of Lactobacillus acidophilus D2/CSL (CECT 4529) supplementation in drinking water on chicken crop and caeca microbiome
Source: PLoS One. 2020 Jan 24;15(1):e0228338. doi: 10.1371/journal.pone.0228338 (PMC6980619; doi:10.1371/journal.pone.0228338)
Supplement: S7 Table — (DOCX) [file pone.0228338.s007.docx]

**S7 Table. P values calculated for the Simpson, Shannon and Pielou indexes quantified for the genera identified in the caeca and crops of chickens belonging to the tested treatments (i.e., day 1, high dose (HD) 14 and 35 days, low dose (LD) 14 and 35 days, control (C) 14 and 35 days).**

|  | C vs LD | C vs HD | LD vs HD |
| --- | --- | --- | --- |
| Caeca 14 d | | | |
| Simpson | 0.760 | 0.760 | 0.933 |
| Shannon | 0.760 | 0.760 | 0.760 |
| Pielou | 0.760 | 0.760 | 0.760 |
| Caeca 35 d | | | |
| Simpson | **0.039** | **0.030** | 0.695 |
| Shannon | 0.370 | 0.398 | 0.695 |
| Pielou | 0.370 | 0.398 | 0.695 |
| Crops 14 d | | | |
| Simpson | 0.235 | 0.452 | 0.695 |
| Shannon | 0.235 | 0.452 | 0.576 |
| Pielou | 0.235 | 0.452 | 0.695 |
| Crops 35 d | | | |
| Simpson | 0.880 | 0.805 | 0.805 |
| Shannon | 0.880 | 0.805 | 0.805 |
| Pielou | 0.880 | 0.805 | 0.805 |
